# ArchAlign: coordinate-free chromatin alignment reveals novel architectures

**DOI:** 10.1186/gb-2010-11-12-r126

**Published:** 2010-12-23

**Authors:** William KM Lai, Michael J Buck

**Affiliations:** 1 Department of Biochemistry and the Center of Excellence in Bioinformatics and Life Sciences, State University of New York at Buffalo, 701 Ellicott St, Buffalo, New York 14203, USA

## Abstract

To facilitate identification and characterization of genomic functional elements, we have developed a chromatin architecture alignment algorithm (ArchAlign). ArchAlign identifies shared chromatin structural patterns from high-resolution chromatin structural datasets derived from next-generation sequencing or tiled microarray approaches for user defined regions of interest. We validated ArchAlign using well characterized functional elements, and used it to explore the chromatin structural architecture at CTCF binding sites in the human genome. ArchAlign is freely available at http://www.acsu.buffalo.edu/~mjbuck/ArchAlign.html.

## Rationale

The development of protein and DNA sequence alignment algorithms in the 1970s and 1980s revolutionized the functional characterization of unknown proteins and genes [1,2]. Since then sequence-based alignments have become so accepted that when a pairwise percentage identity is high enough, a gene or protein is now assigned a function without biochemical confirmation [3]. Similar to the explosion of sequence data in the 1980s, today there is an exponential growth in chromatin structural data. The majority of chromatin data are being generated by next-generation DNA sequencing combined with chromatin immunoprecipitation (ChIP), FAIRE (formaldehyde-assisted isolation of regulatory elements), DNAse I hypersensitivity, or micrococcal nuclease (MNase) digestion assays [4]. Analysis of these high resolution datasets has discovered shared chromatin architectures at previously defined functional elements in the genome; however, identification of new functional elements and their chromatin signatures remains limited.

Currently, the only way to characterize chromatin architecture is to have an accurately mapped functional element in the genome. Functional elements include genes for protein and non-coding RNAs, and regulatory sequences that direct essential functions such as gene expression, DNA replication, and chromosome inheritance. With an accurately mapped functional element, chromatin structural data are aligned by the genomic coordinates and an average profile is created. For example, transcription start sites (TSSs) in *Saccharomyces cerevisiae *have a well documented nucleosome-depleted region approximately 50 to 100 bp upstream of the TSS, flanked by a non-canonical acetylated nucleosome containing the histone variant H2A.Z [5]. Chromatin architecture at these regions was identified because TSSs had been accurately determined through other molecular methods. In addition to TSSs, researchers have used genomic datasets to identify shared chromatin architectures at origins of replication [6], intron-exon junctures [7-11], and enhancers [12]. All successful analyses have started with an accurately mapped functional element, which was used to align all regions containing that functional element. The chromatin architecture was then determined by averaging the chromatin data for aligned regions. For poorly mapped functional elements or elements having an unknown directionality, the chromatin structural profile loses definition and directionality is obscured.

Insulator elements are an example of a genomic element that has not been accurately mapped and has not been extensively characterized. Insulators function to restrict transcriptional enhancers from activating unintended promoters, by acting as a barrier between chromatin contexts [13-15] or by mediating intra- and interchromosomal contacts [16]. While insulators are critical for gene regulation, only a few have been identified [15,17]. A key component of insulators in vertebrates is the ubiquitously expressed CCCTC binding factor (CTCF). The genome-wide binding locations for CTCF have been determined in multiple cell lines by both ChIP-chip and ChIP-seq [18,19] and these locations have been proposed to be insulator sites. Due to limitations in the resolution for all ChIP experiments, the exact site of CTCF binding cannot be determined. In addition, CTCF is part of a multimeric complex that in total defines the location and directionality of insulator elements. Therefore, CTCF binding can only identify insulators within 100 to 200 bp and any directionality within insulators is unknown.

Identification of shared chromatin architecture at functional sites has recently become an active area of research [20-25], but most studies focus on well-defined transcriptional promoters. While these approaches have provided extensive insight into the chromatin architecture at well-defined genomic features, there has been very limited work to identify shared chromatin architectures for unmapped, poorly mapped, or unknown genomic features. Two groups have developed unsupervised approaches to identify overrepresented chromatin states in a genome [24,25]. Hon *et al.*[25] used a variant of a standard motif finding approach with a probabilistic method and were able to uncover 16 distinct signatures and the known patterns at TSSs and enhancers. Ernst and Kellis [24] used a multivariate hidden Markov model to identify how often different chromatin mark combinations are found with one another and used this to identify chromatin states. These two approaches are limited in that while they can identify overrepresented chromatin signatures, they cannot identify less abundant signatures or be used to identify the shared architecture at user-defined regions of interest. To address this limitation, we developed ArchAlign, an algorithm that identifies shared chromatin structural patterns for user-specified regions of interest, from high-resolution chromatin structural datasets derived from next-generation sequencing or tiled microarray approaches. ArchAlign was designed and validated with data from mononucleosomes isolated by MNase digestion [26], and can be used with any dataset that can be converted into high-resolution log ratios. We used ArchAlign to align the nucleosome positions at CTCF binding sites, and uncovered a novel directional chromatin architecture containing positioned H2A.Z nucleosomes with the histone tail modifications H3K4me3, H3K4me2, H3K4me1, H3K9me1, and H3K20me1. These results define a shared structure at many CTCF sites and provide a framework for further exploration of the chromatin structure at insulator elements.

## Results

### ArchAlign design and implementation

**Figure 1:**
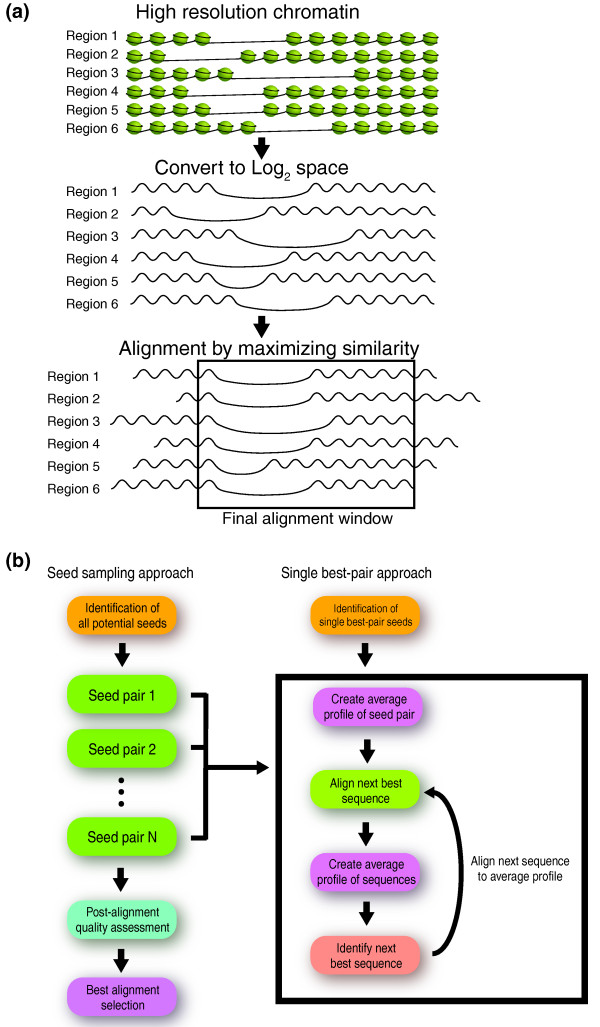
**Overview of the ArchAlign algorithm**. **(a) **High-resolution chromatin structural data from regions of interest are converted into log_2 _ratios and aligned within a user defined alignment frame. **(b) **ArchAlign has two options for aligning chromatin structural patterns: 1, single-best-pair uses the two regions with the highest similarity, by Pearson or Spearman correlation or by Euclidian distance, to seed a single progressive alignment; 2, seed sampling is a more comprehensive search of the possible alignment space, which uses every region within the alignment as a possible seed for an independent alignment. The best alignment is selected as the one that maximizes the correlation across all regions in the alignment frame.

ArchAlign has two methods for aligning regions and three similarity/distance metrics for scoring the similarity between two regions (Figure 1a). Depending on the data type, the user can select between Pearson or Spearman correlation, or Euclidean distance. For all the analysis presented in this manuscript, the Pearson correlation was used as the scoring function. The first alignment method, known as the single-best-pair approach, uses the two regions with the highest similarity as the template pattern to seed the alignment (see Materials and methods). To identify the single-best-pair, every region is compared against every other region at all possible displacements, upstream or downstream, for a predetermined frame size of alignment. For example, using a 1-kb frame of alignment on 200 2-kb regions at 10-bp resolution results in 1,990,000 possible best pairs without reversals and 3,980,000 with reversals. The region reversal option allows the identification of unidirectional chromatin structures, as evident at TSSs, and is advantageous when the actual orientation is unknown. This option compares every possible reversed combination of region frame against every other possible region frame, in addition to its non-reversed comparisons. Once the best pair is identified, an optimal pattern is derived as its average within the alignment frame. The optimal best-pair pattern is then systematically shifted and compared against all remaining regions in the dataset (with optional orientation reversal). The region with the highest similarity is then added to the profile by a weighted average and the process is repeated until all regions have been added to the alignment (Figure 1b).

The second approach, known as seed sampling, is a more comprehensive search of the possible alignment space. Every region in the alignment is used as one-half of the optimal seed pattern for an independent alignment. Therefore, for a dataset with *n *regions, *n *independent alignments are generated as described for the single-best-pair approach (Figure 1b). To determine which of the *n *alignments is the best alignment for the dataset, a post-alignment quality assessment is performed by calculating the average correlation or distance of each aligned region to every other aligned region (see Materials and methods). The alignment that maximizes the similarity across all regions is then selected as the optimal alignment.

### ArchAlign validation

**Figure 2:**
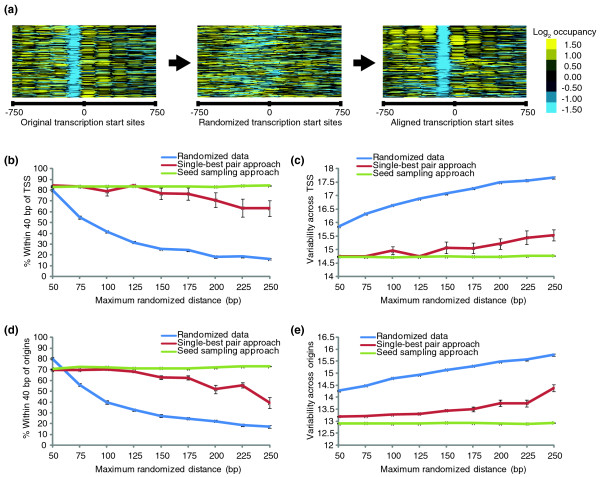
**Validation of ArchAlign. (a) **The location of the TSSs was randomized by 50 to 250 bp for 200 structurally similar TSSs from *S. cerevisiae*, and the nucleosome occupancy data, centered at the randomized TSS coordinates, were aligned with ArchAlign. A total of 1.5 kb surrounding the TSSs is shown with -750 referring to the distance upstream and 750 referring to the distance downstream. **(b) **The TSS data were randomized by shifting the center upstream or downstream from the actual location by a maximum distance of 50 to 250 bp at 25-bp intervals of increasing randomization. The randomized TSS coordinates were then used to generate 1.5-kb regions of nucleosome occupancy at 10-bp resolution surrounding the coordinates (blue line). **(c,e) **Variability for the alignment of TSSs or origins was calculated as the average root of sum of squares for that region compared to the mean profile. Variability for the entire alignment was estimated as the average of all regions. The graphs show the mean and standard error of each of the genomic features' ten alignments' overall variability at each randomization. **(d) **The origins of replication were randomized as described above for TSSs and the randomized coordinates were then used to generate 2-kb regions of nucleosome at 10-bp resolution surrounding the coordinates (blue line). Randomized nucleosome occupancy regions were then entered into ArchAlign using both the single-best-pair (red line) and seed sampling (green line) approach. Each interval of randomization was repeated 10 times for a total of 90 randomized datasets for each genomic feature. ArchAlign's output was then tested for similarity to the original data by determining the percentage of aligned TSSs and origins that were within 40 bp upstream or downstream of their original positions.

ArchAlign was validated by randomizing coordinates for 200 TSSs from *S. cerevisiae *[27], taking the nucleosome occupancy data centered at the randomized TSSs coordinates, and then aligning the randomized data (Figure 2a). Data were randomized by shifting the center upstream or downstream 50 to 250 bases from the actual location. The coordinates of the aligned TSSs were then compared against their original non-randomized TSSs, and accuracy was determined as the percentage of aligned TSSs within 40 bp of their original location (Figure 2b). The overall variability across the alignment was determined as the average sum of squares (Figure 2c). ArchAlign with the single-best pair approach was able to accurately align approximately 80% of the randomized data, when the randomization was less than 150 bp from the original TSSs' coordinates. However, as randomization increased, the overall accuracy of the alignments decreased, with only 60% of the randomized data within 40 bp of their original coordinates, and the variability across the alignment increased in a similar fashion. The decrease in accuracy and the increase in variability for the single-best-pair approach is likely due to poor seed selection since the most similar regions do not necessarily represent the optimal chromatin signature for the entire alignment. ArchAlign with seed sampling produced high-quality alignments regardless of randomization, while maintaining a consistent variability across the alignment. The disadvantage of the seed sampling approach is the computational time involved. Since seed sampling generates one alignment for every region, an alignment of *n *regions requires *n *times as much computational time as the single-best-pair approach.

To ensure that ArchAlign can accurately align chromatin signatures located at various genomic features, we further validated ArchAlign with chromatin data for origins of replications from *S. cerevisiae*. Origins in *S. cerevisiae *have a well-characterized nucleosome-depleted region surrounded on both sides by an array of nucleosomes [28]. We used all origins, 156 of 222, that contained a complete nucleosome occupancy profile identified in the recent study of Berbenetz *et al.*[28]. The origins were then randomized in an identical method to the TSSs and aligned using the same parameters as previously stated. As shown previously with TSSs, ArchAlign using the seed sampling approach was able to produce high quality alignments with low variability regardless of the level of randomization (Figure 2d,e).

### Alignment of nucleosome occupancy at CTCF binding in CD4+ cells

**Figure 3:**
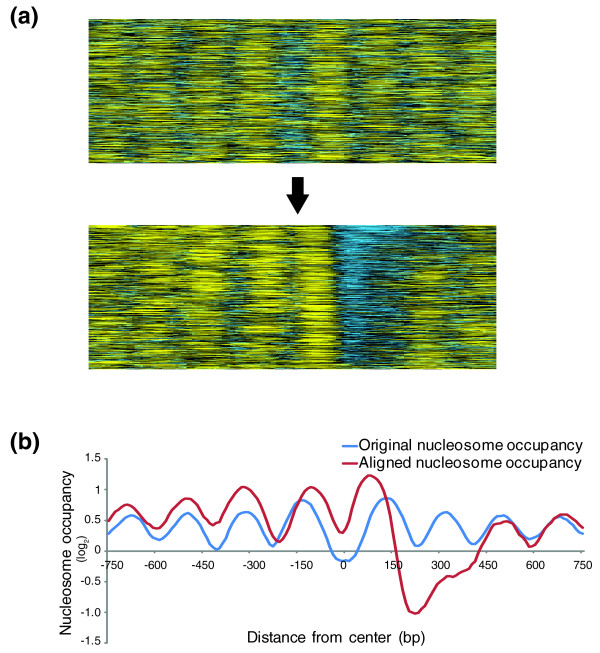
**ArchAlign uncovers an asymmetric nucleosome pattern at CTCF sites. (a) **Nucleosome occupancy profiles were examined at a resolution of 10 bp centered on 1,000 CTCF binding sites in CD4+ cells, and were aligned with ArchAlign with a 1.5-kb alignment frame with reversal of regions enabled [30,37]. **(b) **Comparison of the nucleosome profiles before (blue) and after alignment with ArchAlign (red).

To determine if ArchAlign can uncover novel chromatin architecture, nucleosome occupancy near CTCF binding sites in human CD4+ cells was examined. We selected the top 1,000 CTCF binding sites, which are located at least 2 kb away from a known TSS [29,30]. The nucleosome occupancy data surrounding (±1 kb) each CTCF binding site were extracted and aligned with ArchAlign (Figure 3a). Since directionality of the chromatin structure at CTCF binding sites is unknown, ArchAlign was run allowing region reversal using the seed sampling approach. After alignment, the apparently well-positioned nucleosomes flanking both sides of CTCF binding sites were lost, and a unidirectional nucleosome pattern was observed, with an array of well-positioned, highly occupied nucleosomes on the left of a nucleosome-depleted region (Figure 3b). The directionality discovered after alignment is not a function of asymmetric binding of CTCF [14], since orientation of the nucleosome data by the directionality of the CTCF binding motif still generates a bimodal peak (Figure S1 in Additional file 1). The directional independence of CTCF binding with respect to the nucleosome patterning surrounding it suggests that the primary binding motif of CTCF itself is not responsible for the asymmetrical chromatin signature at these sites. Rather, proteins that interact with CTCF or additional CTCF interactions beyond the consensus motif are driving the directionally observed in the chromatin structure (see Discussion).

### Identification of epigenetic architecture using the ArchAlign coordinates

**Figure 4:**
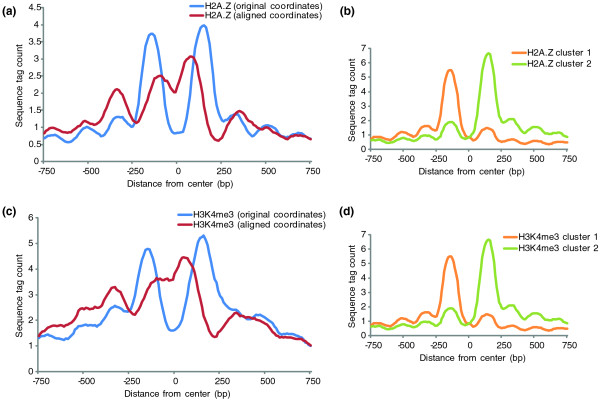
**Effect of nucleosome alignment on CTCF correlated histone modifications**. **(a,c) **Plots of the average tag counts at the unaligned and aligned CTCF sites for (a) H2A.Z and (c) H3K4me3. **(b,d) **Sequence tag data for H2A.Z at CTCF sites was clustered into two groups by *k*-means clustering after centering each region by average tag count [39]. The average profiles for the two groups for (b) H2A.Z and (d) H3K4me3 were plotted.

To determine if alignment of the nucleosomes at CTCF sites revealed unique chromatin architecture, we examined the available histone variant and histone modification data for human CD4+ cells at these sites [12,30]. The histone variant H2A.Z and the histone tail modification H3K4me3 were specifically examined due to their previously characterized correlation to CTCF binding [13,14,30]. As previously shown for unaligned CTCF sites, there was a bimodal peak surrounding the CTCF binding site for both H2A.Z and H3K4me3 (Figure 4a,c). The coordinates derived from the alignment of nucleosomes surrounding CTCF were used to generate the aligned profiles of all histone modifications. When we generated average profiles for H2A.Z and H3K4me3, the bimodal distribution was lost, and there were three ordered H2A.Z and H3K4me3 nucleosomes to the left of the nucleosome-depleted region. These results suggest that the bimodal peaks of H2A.Z and H3K4me3 seen in previous publications are likely an artifact due to averaging of all CTCF binding sites without considering the asymmetry/orientation of the chromatin structure [14,30]. To confirm that the original data did in fact contain two groups with reverse orientations and only a single peak of H2A.Z at individual CTCF sites, we clustered the examined regions by H2A.Z sequence tag counts into two groups (Figure 4b,d). Two distinct and opposite peaks were identified in both cases, which provides further evidence that the directionality proposed by ArchAlign is valid. We further explored the chromatin architecture at CTCF sites by mapping all available histone modification datasets from CD4+ cells [12,30]. In addition to H2A.Z and H3K4me3, H3K4me2, H3K4me1, H3K9me1, and H4K20me1 displayed a similar directional pattern (Figure S2 in Additional file 1), while other methylation and acetylation marks were not associated with CTCF sites (Figures S3 and S4 in Additional file 1). To confirm that the discovered structure at CTCF sites is specific for CTCF, we randomly selected 1,000 2-kb regions from the genome and aligned the nucleosome occupancy data using the same ArchAlign parameters as before (Figure S5 in Additional file 1). For all randomizations, there was an absence of a chromatin architecture after alignment, confirming that the discovered architecture at CTCF sites is specific to these regions.

ArchAlign was able to uncover the unique chromatin architecture located at CTCF sites using only nucleosome occupancy data. The identified architecture contains a nucleosome-depleted region located near CTCF binding sites with adjacent positioned nucleosomes. Overlaying the histone modification and variant data using the aligned coordinates on top of the nucleosome occupancy showed a strong preference for the presence of the H2A.Z nucleosome variant on the strongly positioned nucleosomes as well as the concurrent presence of the histone tail modifications H3K4me3, H3K4me2, H3K4me1, H3K9me1, and H4K20me1. These results suggest that CTCF is a component of direction-dependent chromatin architecture at the majority of its binding sites, which may be functionally important for its role as an insulator.

## Discussion

Insulator elements have been characterized by their ability to act as a barrier between differing chromatin contexts [13,16]. We found a directional chromatin signature at CTCF sites that appears similar to a barrier between chromatin contexts. On one side there are H2A.Z nucleosomes with H3K4, H3K9, and H3K20 methylation and on the other side there is a reduced nucleosome occupancy. The discovered asymmetric architecture at CTCF appears similar to the recent association of CTCF at borders between repressive chromatin marks [13,30], or heterochromatic lamina associated domains [31]. The functional importance of the chromatin architecture and polarity for insulators has yet to be determined, but our alignment will act as a guide for future functional dissections.

CTCF binding location alone is not capable of providing an accurate alignment because the chromatin architecture at CTCF insulators is likely caused by other associated proteins, the underlying DNA sequence at the region, or a combination of both. At the well-studied H19 imprinting control region nucleosome positioning has been shown to be regulated by the underlying DNA sequence, not CTCF binding [32]. In addition, CTCF is known to interact with multiple DNA binding transcription factors, chromatin modifying proteins, and nuclear architectural proteins [33]. Therefore, it is likely that CTCF is only a single component of a multimeric complex located at insulators and that this insulator complex with the underlying DNA sequence defines the chromatin architecture at insulators. Identifying the shared chromatin architecture at insulators using CTCF binding location is analogous to identifying the shared architecture at TSSs using only the binding location of a transcription factor. To illustrate this concept, we examined the shared chromatin architecture around the binding sites for three abundant yeast transcription factors (Figure S6 in Additional file 1). The average nucleosome occupancy profile of the transcription factor binding site was compared before and after alignment with ArchAlign to the average nucleosome profile of the TSSs adjacent to the binding site. Since most transcription factors can bind in either orientation in relationship to TSSs and at various distances, the profile derived by only the binding site appears symmetrical and not well resolved. After alignment with ArchAlign the aligned profile has a similarity to the TSSs' derived profile and the true asymmetric nature is uncovered.

Similar to DNA and protein sequence alignment algorithms, ArchAlign does not identify the base pair location for a feature of interest. To identify the location of a genomic feature with ArchAlign, example regions containing an experimentally mapped feature need to be included in the alignment. The location of the unknown features can then be inferred from the alignment. The accuracy of the alignment is dependent on both the accuracy of the example regions and the extent to which the chromatin is organized around that feature. As evident from TSSs and CTCF binding sites, histone variants and histone tail modifications help define distinct chromatin architecture for certain genomic features. Future versions of ArchAlign will incorporate these datasets in order to produce an even more biologically relevant alignment.

ArchAlign is the first tool developed to align chromatin structural data and will prove highly valuable for analyzing chromatin datasets from genomes lacking substantial genomic feature annotation. Currently, there are many genomic features that cannot be accurately mapped by available techniques. For example, TSSs in *Caenorhabditis elegans *are difficult to map due to trans-splicing of the majority of mRNAs, which causes the 5' ends of different messages to have the same leader sequence [34], and origins of replication in *Schizosaccharomyces pombe *are difficult to map accurately, because *S. pombe*'s origin recognition complex does not bind to specific DNA sequences but to AT-rich regions [35]. ArchAlign requires only the general coordinates of a feature in order to determine the likely structural pattern present around it. In addition, as demonstrated for CTCF sites, even accurately mapped features may have a previously unrecognized directionality obscuring results that could be revealed by alignment with ArchAlign.

## Materials and methods

### *S. cerevisiae *nucleosome occupancy

*S. cerevisiae *genome-wide nucleosome occupancy maps were downloaded from the Segal Lab and transformed into a log_2 _ratio for each base pair [26]. Mapped sequence tags from a MNase digestion were extended to the average sequence length for that experiment (150 to 200 bp) and normalized nucleosome occupancy at every base pair was determined as the log-ratio between the number of reads that cover that base pair and the average number of reads per base pair across the genome [26]. The 200 TSSs used in validation were randomly selected from the previously defined cluster 3 of similar TSSs exhibiting high expression levels and a similar nucleosome occupancy pattern [27]. The 156 origins of replication used for validation were selected from the original list of 222 characterized origins because they contained no gaps in the nucleosome occupancy dataset [28]. The 70 MBP1, 76 GCN4, and 63 SWI4 binding sites identified by ChIP-chip experiments were selected from the original list of 127 MBP1, 107 GCN4, and 145 SWI4 sites because they contained no gaps in nucleosome occupancy [36].

### Human CD4+ resting cells

#### Nucleosome occupancy

Genome-wide nucleosome occupancy maps for CD4+ cells were downloaded from NCBI [37] and transformed into a log_2 _ratio, as described above, using a tag extension of 120 bp.

#### CTCF binding sites

CTCF binding data were downloaded from NCBI [30]. The top 1,000 CTCF sites were selected by running a MACS analysis [29] and identifying the highest peaks determined by fold enrichment of CTCF binding in the genome. The coordinates for the top 10,000 peaks were then used to generate the nucleosome occupancy profiles in the log_2 _occupancy dataset previously generated. All CTCF sites for which complete data were not available or were within 2 kb of TSSs of a known gene were removed from the original set of sites. The remaining top 1,000 sites by fold enrichment were then selected as CTCF binding sites.

#### CD4+ histone modification maps

CD4+ histone modification data were downloaded from NCBI [12,30]. Genome-wide maps of sequence tag count were then generated for all datasets assuming an extension length of 120 bp as previously described.

#### Random regions

A random number generator was used to generate the genomic coordinates for 10,000 random non-overlapping regions. The data were then extracted as previously described. The first 1,000 remaining regions after filtering were then selected as the random regions.

### ArchAlign

#### Scheme 1: overview of chosen seed alignment

Usage: Chosen Seed Alignment (X, Y), where X = Seed Region 1, Y = Seed Region 2, Z = List of Regions, n = Number of Regions, w = Optimal Window of Region, and P = Average Profile.

Identify windows of X and Y that maximize the similarity

$$\text{P}=\left({\text{X}}_{\text{w}}+{\text{Y}}_{\text{w}}\right)/\text{2}$$

Repeat for i = 3 to n

Repeat for j = 0 to Length of Remaining Regions

Identify window of Z_j _that maximizes similarity to P

Identify Region j that contained the window that produced the highest similarity to P

$$\text{P}=\left(\text{P}\times \left(\text{i}-\text{1}\right)+{\text{Z}}_{\text{j};\text{w}}\right)/\text{i}$$

Remove Z_j _from Z

Output Optimal Windows of All Regions

#### Scheme 2: Overview of single best-pair alignment

Usage: Single Best-Pair Alignment(Z)

Repeat for i = 0 to n

Repeat for j = 0 to n

Identify windows of Z_i _and Z_j _that maximize similarity out of all possible regions given i ≠ j

Identify which two Regions contained the windows that produced the highest similarity to each other for Z_i _and Z_j_

Chosen Seed Alignment(Z_i_, Z_j_)

#### Scheme 3: Overview of seed selection alignment

Usage: Seed Selection Alignment(Z), where A_i _= Alignment with a forced seed from Region i.

Repeat for i = 0 to n

Repeat for j = 0 to n

Identify windows of Z_i _and Z_j _that maximize similarity out of all possible regions given i ≠ j

Identify which two Regions contained the windows that produced the highest similarity to each other for Z_i _and Z_j_

A_i _= Chosen Seed Alignment(Z_i_, Z_j_)

Post-Alignment Quality Assessment(A_i_)

Identify and output alignment that produced the highest Post-Alignment Quality Assessment

#### Equation for post-alignment quality assessment

Usage: Post-Alignment Quality Assessment(A), where A = Alignment.

$$\frac{\sum_{X=0}^{n}\sum_{Y=0}^{n}SimilarityMetric(A_{X},A_{Y})I_{\left(X\neq Y\right)}I_{\left((X-Y)\geq 1\right)}}{\sum_{X=1}^{n-1}X}$$

#### Compiled

ArchAlign was designed and written in C++ then compiled and run on a 64-bit Linux machine with 8 × 2.76 GHz Xeon X5550 cores, 48 GB RAM, and 80 TB attached disk storage array. The current version of ArchAlign is designed to use only a single CPU core per run.

#### Validation

The original (±750 bp) nucleosome profiles of the 200 TSSs were extracted at a resolution of 10 bp. ArchAlign was performed with a sliding window of 1 kb with region reversal disabled. The original (±1 kb) nucleosome of 156 origins of replication were extracted at a resolution of 10 bp. ArchAlign was then performed with a sliding window of 1.5 kb with region reversal disabled.

#### CTCF

The (±1 kb) nucleosome profiles of the top 1,000 were extracted at a resolution of 10 bp and ArchAlign was run with a sliding window of 1.5 kb with region reversal enabled.

#### Random region

The (±1 kb) nucleosome profiles of 1,000 random regions were extracted at a resolution of 10 bp and ArchAlign was run with a sliding window of 1.5 kb with region reversal enabled.

#### Run times

Alignment using seed sampling without reversals of 200 2-kb regions at 10-bp resolution with a sliding window of 1.5 kb requires less than 5 minutes of CPU time. Alignment using seed sampling with reversals of 1,000 2-kb regions at 10-bp resolution with a sliding window of 1.5 kb requires approximately 20 hours of CPU time. Increases in number of regions, region size, data resolution, and decreases in window size will result in increases of CPU run time. Alignment using the single-best-pair approach is significantly faster; for a 1,000 2-kb region with reversals at 10-bp resolution with a 1.5-kb window requires less than 10 minutes CPU time.

#### Availability

ArchAlign is available at [38].

## Abbreviations

bp: base pair; ChIP: chromatin immunoprecipitation; CTCF: CCCTC binding factor; MNase: micrococcal nuclease; TSS: transcription start site.

## Competing interests

The authors declare that they have no competing interests.

## Authors' contributions

WKML and MJB conceived and designed the method and wrote the manuscript. WKML implemented ArchAlign.

## Supplementary Material

Additional file 1 **Supplementary figures S1, S2, S3, S4, S5, and S6**. Click here for file
