# Supplementary material for: ArchAlign: coordinate-free chromatin alignment reveals novel architectures
Source: Genome Biol. 2010 Dec 23;11(12):R126. doi: 10.1186/gb-2010-11-12-r126 (PMC3046486; doi:10.1186/gb-2010-11-12-r126)
Supplement: Additional file 1 — Supplementary figures S1, S2, S3, S4, S5, and S6. [file gb-2010-11-12-r126-S1.pdf]

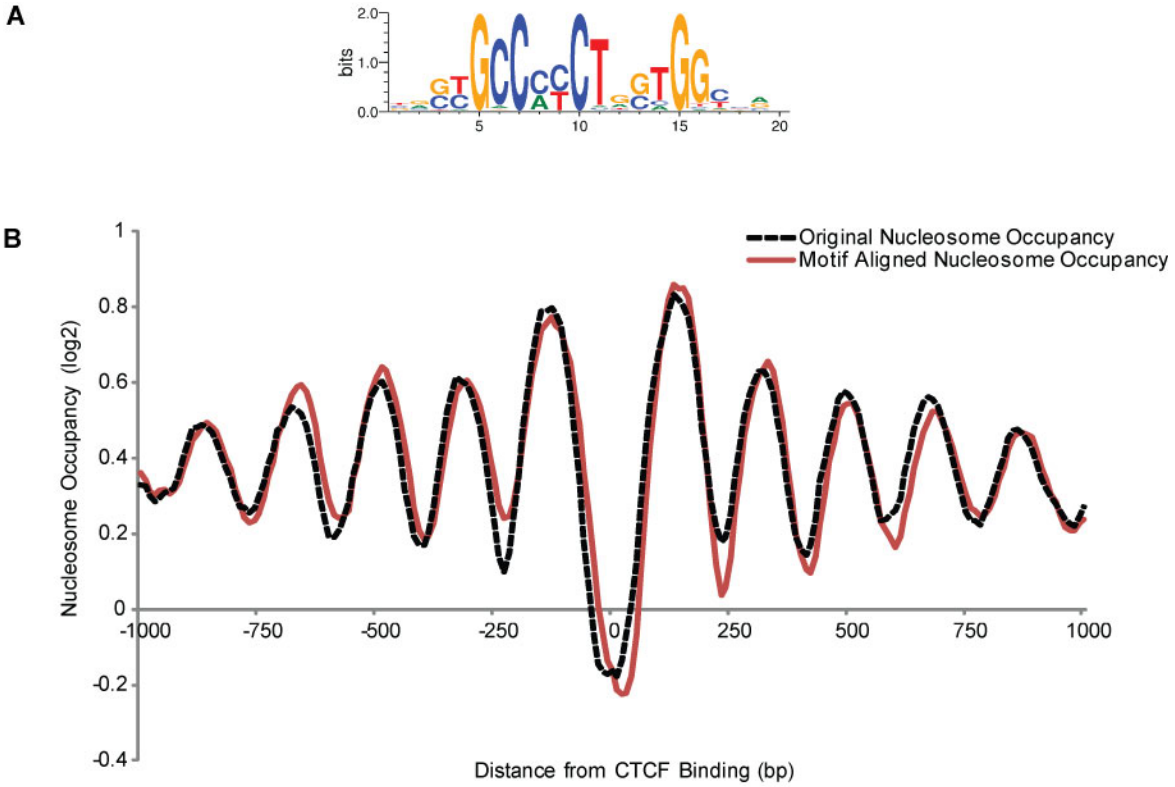

Figure S1: Effect of Motif Orientation on Nucleosome Occupancy. (A) Derived binding motif of CTCF from 6432 occupied sites [1-2]. (B) Nucleosome occupancy profiles were examined at a resolution of 10bp centered on 1,117 CTCF binding sites in CD4+ cells for which data was available and were known to be occupied.

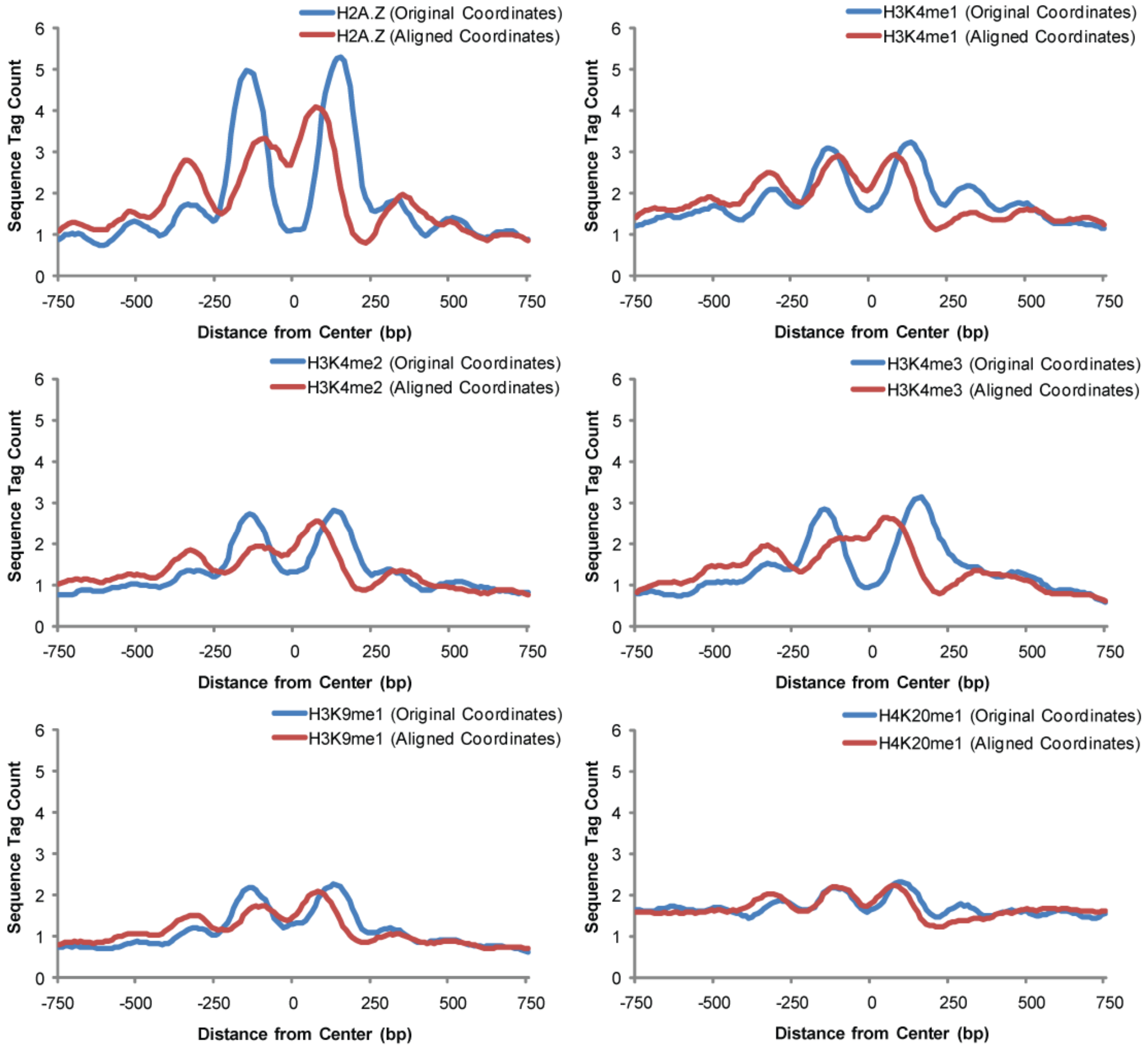

Figure S2: Histone Modifications Associated with CTCF sites. Comparison of histone modifications associated with CTCF binding before (blue) and after alignment (red). Modification data was standardized to a total tag count of 10 million reads per experiment to allow comparisons between different histone modifications.

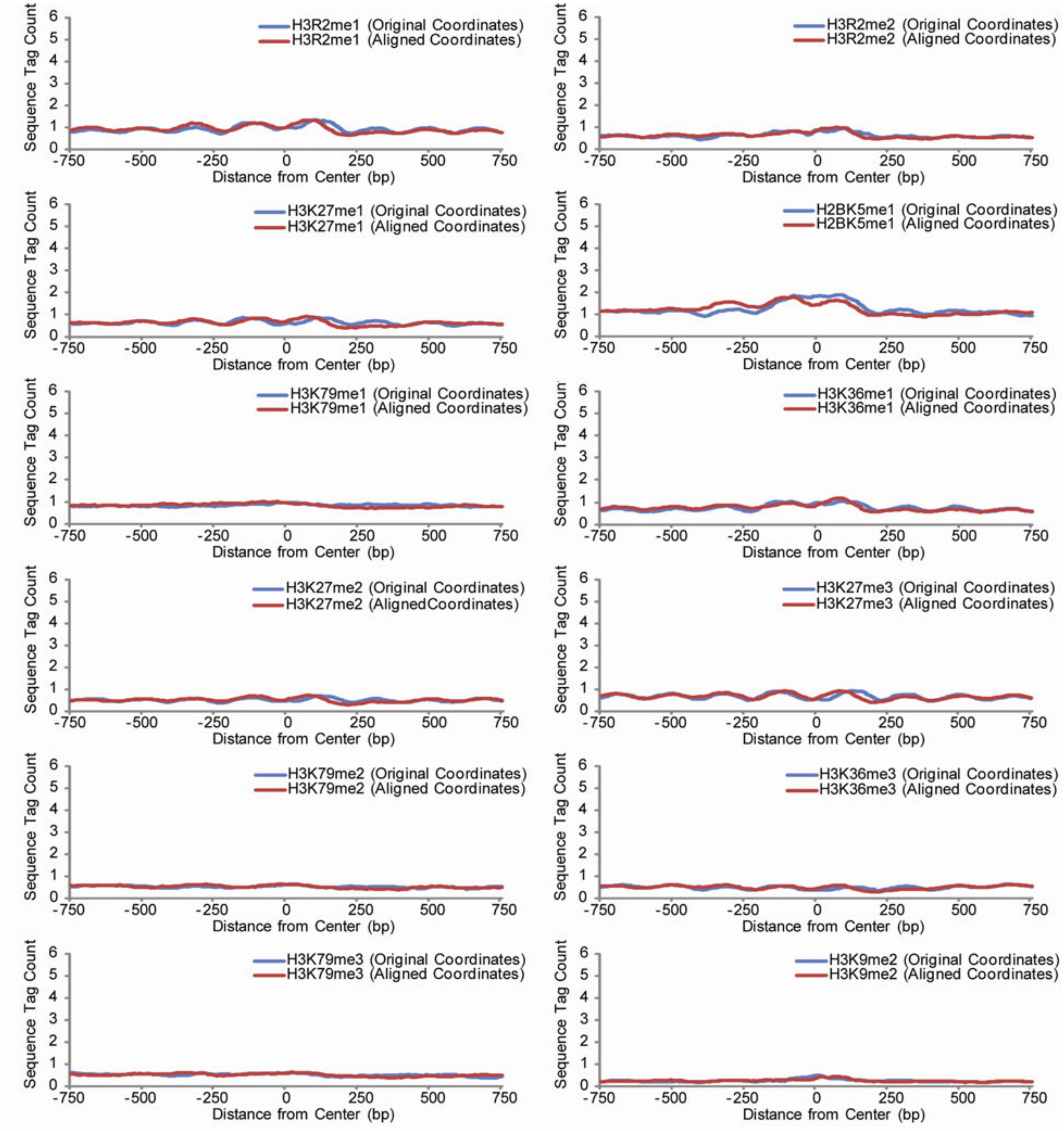

Figure S3: Unassociated Histone Methylation Modifications. Comparison of histone methylation patterns to CTCF binding before (blue) and after alignment (red). Modification data was standardized to a total tag count of 10 million reads per experiment to allow comparisons between different histone modifications.

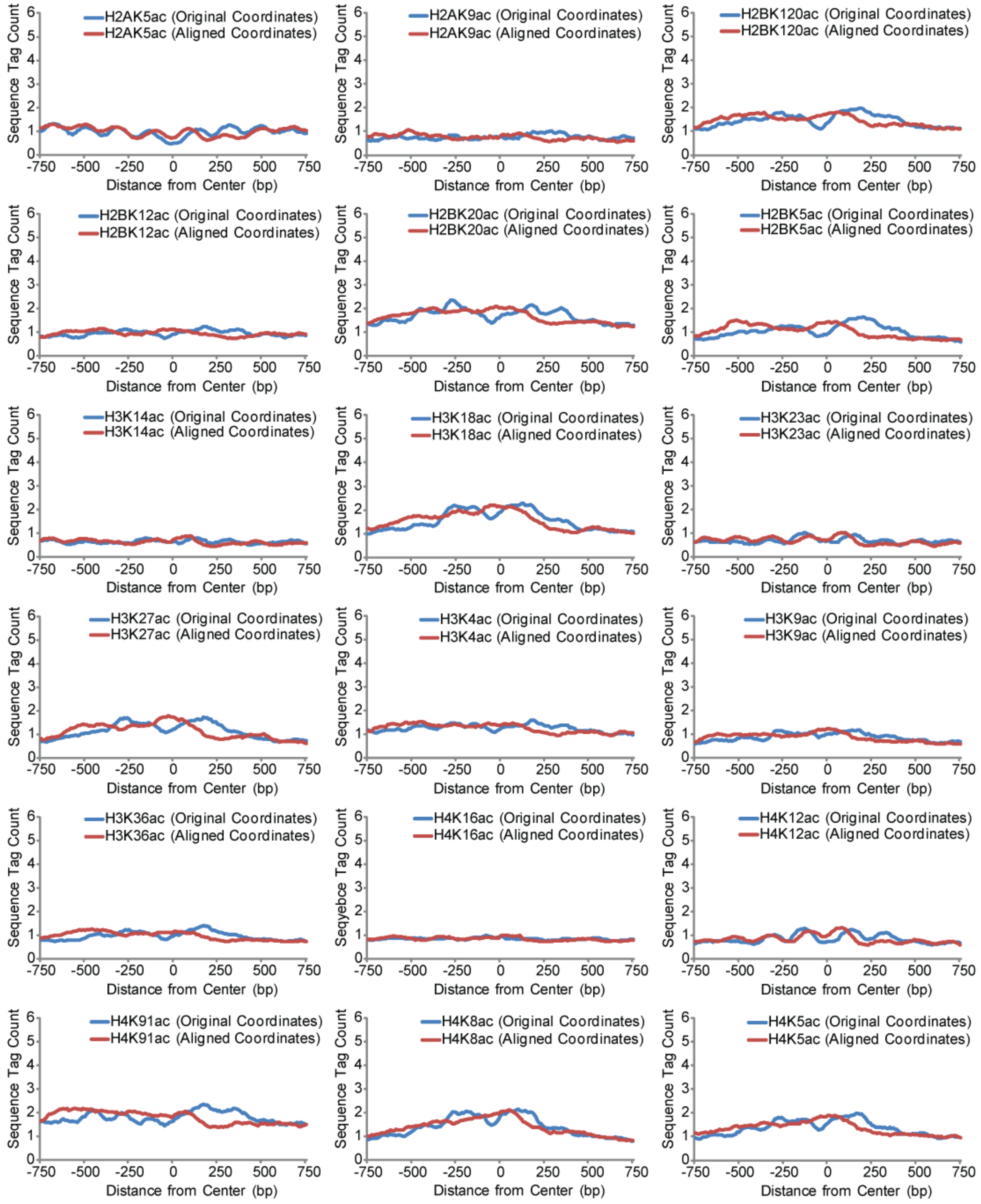

Figure S4: Unassociated Histone Acetylation Modifications. Comparison of histone acetylation patterns to CTCF binding before (blue) and after alignment (red). Modification data was standardized to a total tag count of 10 million reads per experiment to allow comparisons between different histone modifications.

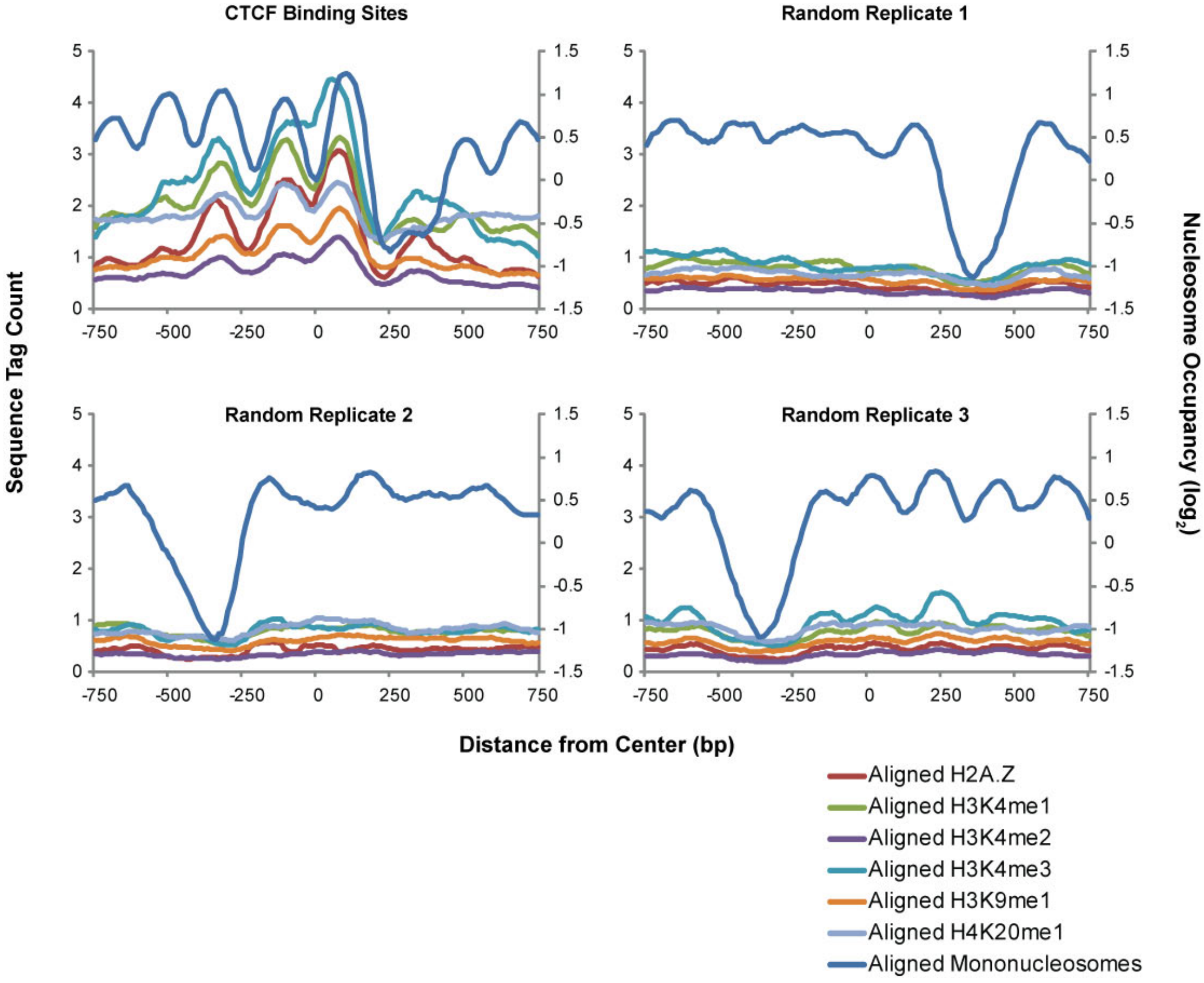

Figure S5: ArchAlign uncovers chromatin signature unique to CTCF sites. Comparison of 1000 CTCF aligned mononucleosomes and associated histone modifications to 3 replicates of 1000 aligned random mononucleosome regions and CTCF associated histone modifications. Average nucleosome occupancy data is plotted as $\log_2$ ratio on the right y-axis, and histone modifications or histone variants are plotted as average sequence tag count on the left x-axis.

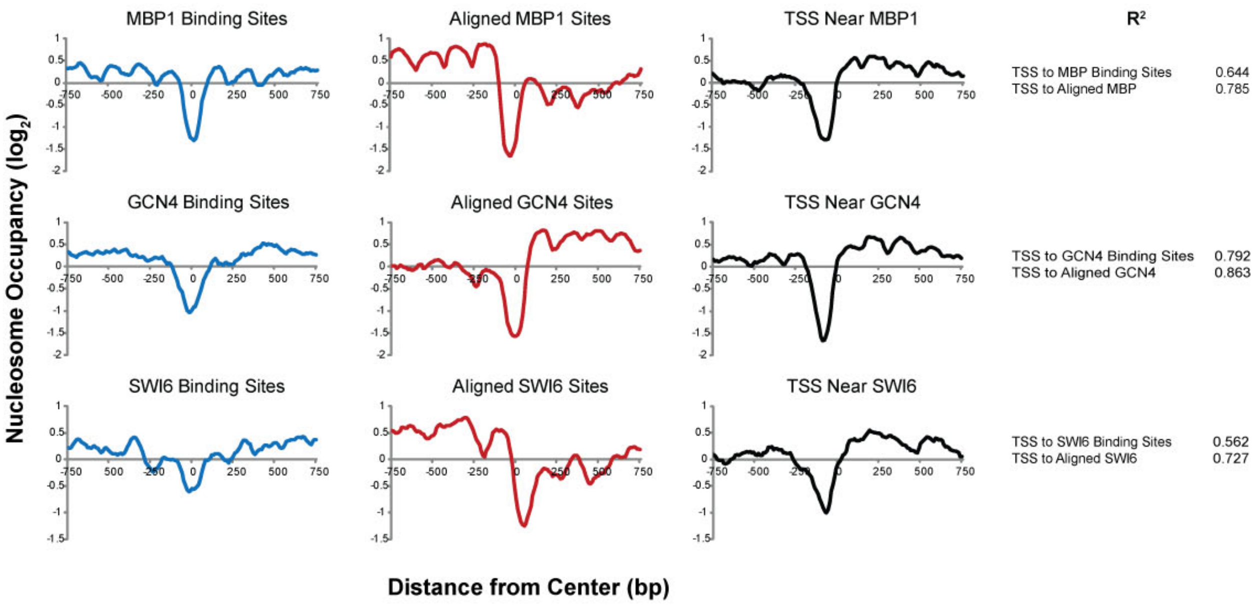

Figure S6: Alignment of Transcription Factor binding sites reveals TSS profile. Average nucleosome profile surrounding binding sites of MBP1, GCN4, and SWI6 (blue) compared to ArchAligned profile (red), and TSSs adjacent to binding sites (black). The Pearson Correlation R^2 was determined by first shifting each profile so that their minima are aligned and reversing profiles when needed.

1. Fu Y, Sinha M, Peterson CL, Weng Z: **The insulator binding protein CTCF positions 20 nucleosomes around its binding sites across the human genome**. *PLoS Genet* 2008, **4**(7):e1000138.
2. Crooks GE, Hon G, Chandonia JM, Brenner SE: **WebLogo: a sequence logo generator**. *Genome Res* 2004, **14**(6):1188-1190.
